# In this Issue

**Published:** 2008

## WHO IS AT RISK? POPULATION CHARACTERIZATION OF ALCOHOL SELF-ADMINISTRATION IN NONHUMAN PRIMATES HELPS IDENTIFY PATHWAYS TO DEPENDENCE

Alcohol abuse and dependence are uniquely human disorders. However, scientists sometimes use animal models to study different aspects of alcohol abuse and dependence, including aspects that cannot be easily or ethically studied in humans. For example, studies of nonhuman primates, who share many genetic, anatomical, physiological, and behavioral characteristics with humans, can help to uncover risk factors that may either predispose a person to alcoholism or accelerate the course of alcohol addiction; these models therefore can shed light on these processes in humans in a way that other animal models cannot. In this article, Dr. Kathleen A. Grant, Mr. James Stafford, Ms. Allison Thiede, Ms. Caitlin Kiley, Ms. Misa Odagiri, and Dr. Betsy Ferguson present findings of studies analyzing drinking behaviors in populations of nonhuman primates and the neurological factors that may underlie differences in alcohol intake levels. The authors also discuss how these findings can help scientists to prevent and treat alcoholism in humans. (pp. 289–297)

## THE MOLECULAR BASIS OF TOLERANCE

Tolerance is the body’s diminished response to alcohol or other drugs over the course of time and repeated exposure. This article by Drs. Andrzej Z. Pietrzykowski and Steven N. Treistman discusses the various degrees of tolerance—acute, rapid, or chronic—and how changes in tolerance induced by alcohol may affect several processes at the molecular, cellular, or behavioral level that are related to the onset of acute, rapid, or chronic tolerance. (pp. 298–309)

## HOW ADAPTATION OF THE BRAIN TO ALCOHOL LEADS TO DEPENDENCE: A PHARMACOLOGICAL PERSPECTIVE

Alcohol causes both short- and long-term changes in brain chemistry. Long-term changes in some of the brain’s signaling systems have been linked to the behavioral effects of alcoholism. If a heavy drinker stops drinking suddenly, these changes can lead to the syndrome of alcohol withdrawal; additionally, they can make an alcohol-dependent person who has stopped drinking more likely to relapse. As described by Drs. Peter Clapp, Sanjiv V. Bhave, and Paula L. Hoffman, many brain chemicals are affected by both chronic exposure to alcohol and sudden cessation of alcohol consumption. Both alcohol-induced and withdrawal-induced changes to these signaling systems may lead to behavioral effects such as reinforcement, enhanced anxiety, and increased sensitivity to stress. The authors also explain how certain brain chemicals can be targeted by drugs meant to treat alcohol dependence. (pp. 310–339)

## FROM ACTIONS TO HABITS: NEUROADAPTATIONS LEADING TO DEPENDENCE

Overlapping cerebral networks associated with behavioral control processes such as reward-guided Pavlovian conditional responses, goal-directed instrumental actions, and stimulus-driven habits can be altered by alcohol and other drug abuse and therefore are important to the study of substance abuse. In this article, Dr. Henry H. Yin describes these networks and the mechanisms underlying reward-guided action selection and discusses their implications for alcohol addiction. (pp. 340–344)

## ALCOHOL DEPENDENCE, WITHDRAWAL, AND RELAPSE

Alcohol dependence is associated with the development of physical and psychological withdrawal symptoms when alcohol use is stopped or significantly reduced. According to Dr. Howard C. Becker, fear of these withdrawal symptoms can perpetuate alcohol abuse in many alcohol-dependent people; moreover, the presence of such symptoms can trigger relapse in those who have abstained from alcohol. As Dr. Becker reports, both clinical studies and basic research studies using animal and human models have demonstrated that alcohol-related (conditioned) cues and contexts as well as stressful stimuli and events can trigger relapse. Improved understanding of the processes contributing to withdrawal and relapse may aid in the development of medications to treat alcohol dependence more effectively. (pp. 348–361)

## MAGNETIC RESONANCE IMAGING OF THE LIVING BRAIN: EVIDENCE FOR BRAIN DEGENERATION AMONG ALCOHOLICS AND RECOVERY WITH ABSTINENCE

Magnetic resonance imaging, or MRI, is a safe and noninvasive technology that allows scientists to examine the brain’s structure and function in real time. MRI technologies can show how the brain changes when exposed to alcohol over the short and long term, as well as how long these changes persist after a person has stopped drinking. In this article Ms. Margaret J. Rosenbloom and Dr. Adolf Pfefferbaum discuss how MRI studies, including longitudinal studies of animal models of alcoholism, can help researchers to understand how alcoholism develops and how it affects the brain. Through these studies, scientists have been able to explore specific alcohol-related changes to the brain, their duration, their effects on behavior, and the mechanisms through which the brain compensates for these changes. These studies suggest that some changes are reversed with abstinence, while others endure long after a person has stopped drinking. (pp. 362–376)

## ALCOHOL-RELATED NEURODEGENERATION AND RECOVERY: MECHANISMS FROM ANIMAL MODELS

Animal studies have established that alcohol can cause damage to brain cells, resulting in the loss of structure and function as well as inhibiting the production of neurons (i.e., neurogenesis), which ultimately leads to cognitive impairment. This article by Dr. Fulton T. Crews describes models of binge alcohol consumption in rats that induce changes in cognition similar to those found in human alcoholics. Findings from these animal studies provide insight into when, where, and how alcohol abuse and abstinence recovery dynamically change brain-cell composition, which could lead to new potential therapies for neurodegeneration, mental diseases, and alcohol use disorders. (pp. 377–388)

## THE ROLE OF SELECTED FACTORS IN THE DEVELOPMENT AND CONSEQUENCES OF ALCOHOL DEPENDENCE

Studying the risk for developing alcoholism and the negative consequences of alcohol dependence is a complex process that requires an understanding of the various factors that may determine the degree of that risk. In this article, Rebecca Gilbertson, Robert Prather, and Dr. Sara Jo Nixon examine how gender, family history, comorbid psychiatric and substance use disorders, and age interact to influence an individual’s risk for alcoholism as well as how they interact with alcoholism to influence neurocognitive functioning following detoxification. (pp. 389–399)

## TREATMENT IMPLICATIONS: USING NEUROSCIENCE TO GUIDE THE DEVELOPMENT OF NEW PHARMACOTHERAPIES FOR ALCOHOLISM

Developing new medications to treat alcohol dependence requires a better understanding of the neuroscience of alcohol-drinking behavior. Although there are currently three approved medications for treating alcohol dependence—disulfiram, naltrexone, and acamprosate—new medications that target different neurochemical systems and that could be used either as adjunctive treatments or to treat subpopulations of drinkers are needed. In this article, Drs. Suchitra Krishnan-Sarin, Stephanie O’Malley, and John H. Krystal discuss the complex neuropharmacology of alcohol and how it can affect many different brain neurotransmitter systems. (pp. 400–407)

